# ECDC’s latest publications

**DOI:** 10.2807/1560-7917.ES.2016.21.35.30327

**Published:** 2016-09-01

**Authors:** Eurosurveillance editorial team

**Affiliations:** 1 European Centre for Disease Prevention and Control (ECDC), Stockholm, Sweden

**Keywords:** public health

## Rapid risk and outbreak assessments

Date of publication: 8 August 2016

### 

#### Enterovirus detections associated with severe neurological symptoms in children and adults in European countries

http://ecdc.europa.eu/en/publications/Publications/01-08-2016-RRA-Enterovirus%2071-Spain,%20France,%20Netherlands.pdf

## Corporate publications

Date of publication: 5 August 2016

### 

#### Achievements, challenges and major outputs 2015: Highlights from the Annual Report of the Director

http://ecdc.europa.eu/en/publications/_layouts/forms/Publication_DispForm.aspx?List=4f55ad51-4aed-4d32-b960-af70113dbb90&ID=1545

## Scientific advice

Date of publication: 15 August 2016

### 

#### Expert opinion on whole genome sequencing for public health surveillance

http://ecdc.europa.eu/en/publications/Publications/whole-genome-sequencing-for-public-health-surveillance.pdf

## Technical report

Date of publication: 29 August 2016

### 

#### EU Laboratory Capability Monitoring System (EULabCap): Report on 2014 survey of EU/EEA country capabilities and capacities 

http://ecdc.europa.eu/en/publications/Publications/laboratory-capability-monitoring-2014-eu-labcap.pdf

Date of publication: 19 August 2016

#### Seventh external quality assessment for *Salmonella* typing

http://ecdc.europa.eu/en/publications/Publications/salmonella-typing-seventh-external-quality-assessment.pdf

Date of publication: 10 August 2016

#### ECDC roadmap for integration of molecular typing and genomic typing into European-level surveillance and epidemic preparedness – Version 2.1, 2016-19

http://ecdc.europa.eu/en/publications/Publications/molecular-typing-EU-surveillance-epidemic-preparedness-2016-19-roadmap.pdf

Date of publication: 10 August 2016

#### ECDC roadmap for integration of molecular typing into European-level surveillance and epidemic preparedness – Version 1.2, 2013

http://ecdc.europa.eu/en/publications/Publications/molecular-typing-EU-surveillance-epidemic-preparedness-2013.pdf ****

## Surveillance reports

Date of publication: 31 August 2016

### 

#### Gonococcal antimicrobial susceptibility surveillance in Europe, 2014

http://ecdc.europa.eu/en/publications/Publications/gonococcal-antimicrobial-susceptibility-surveillance-Europe-2014.pdf

Date of publication: 9 August 2016

#### Influenza virus characterisation, Summary Europe, July 2016

http://ecdc.europa.eu/en/publications/Publications/influenza-virus-characterisation-july-2016.pdf

Date of publication: every Friday

#### ECDC communicable disease threats report

http://ecdc.europa.eu/en/publications/surveillance_reports/Communicable-Disease-Threats-Report/Pages/default.aspx

